# Supplementary material for: A plasmid-encoded peptide from Staphylococcus aureus induces anti-myeloperoxidase nephritogenic autoimmunity
Source: Nat Commun. 2019 Jul 29;10:3392. doi: 10.1038/s41467-019-11255-0 (PMC6662820; doi:10.1038/s41467-019-11255-0)
Supplement: Supplementary file 1 — Supplementary Information File [file 41467_2019_11255_MOESM1_ESM.docx]

**Supplementary Information**

For: Ooi JD et al. A *Staphylococcal* plasmid-derived peptide induces anti-myeloperoxidase nephritogenic autoimmunity

**Supplementary Figure 1**

**
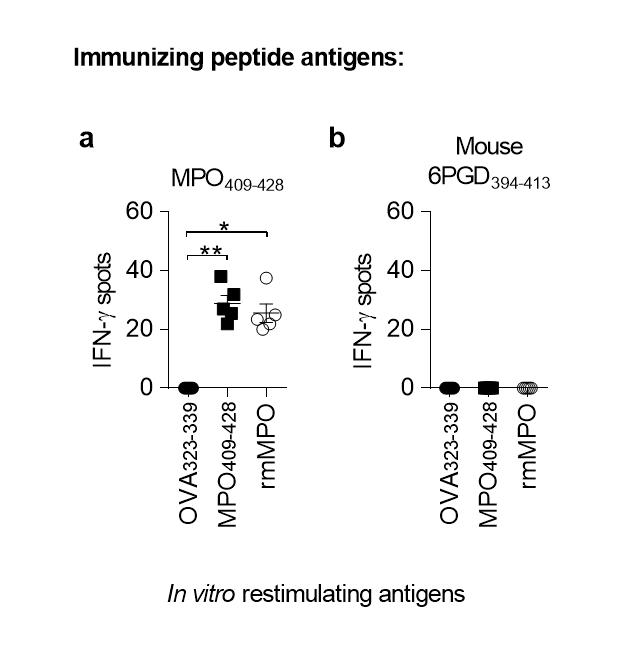
**

**Supplementary Figure 1. The mammalian orthologue of *S. aureus* pSJH101 6PGD_391-410_ does not induce anti-MPO cross reactivity.** C57BL/6 mice (*n* = 5 each group) were immunized with either **a** MPO_409-428_ (positive control PRWNGEKLYQEARKIVGAMV) or **b** mouse 6PGD_394-413_ (FFKSAVDNCQDSWRRVISTGV), then T cell recall responses measured *ex vivo* to OVA_323-339_, MPO_409-428_ or recombinant mouse MPO (rmMPO) by IFN-γ ELISPOT. Each dot represents the response from an individual mouse. Error bars represent the mean ± s.e.m. **P* < 0.01, ***P* < 0.001 by Kruskal-Wallis test. Source data are provided as a Source Data file.

**Supplementary Figure 2**

**
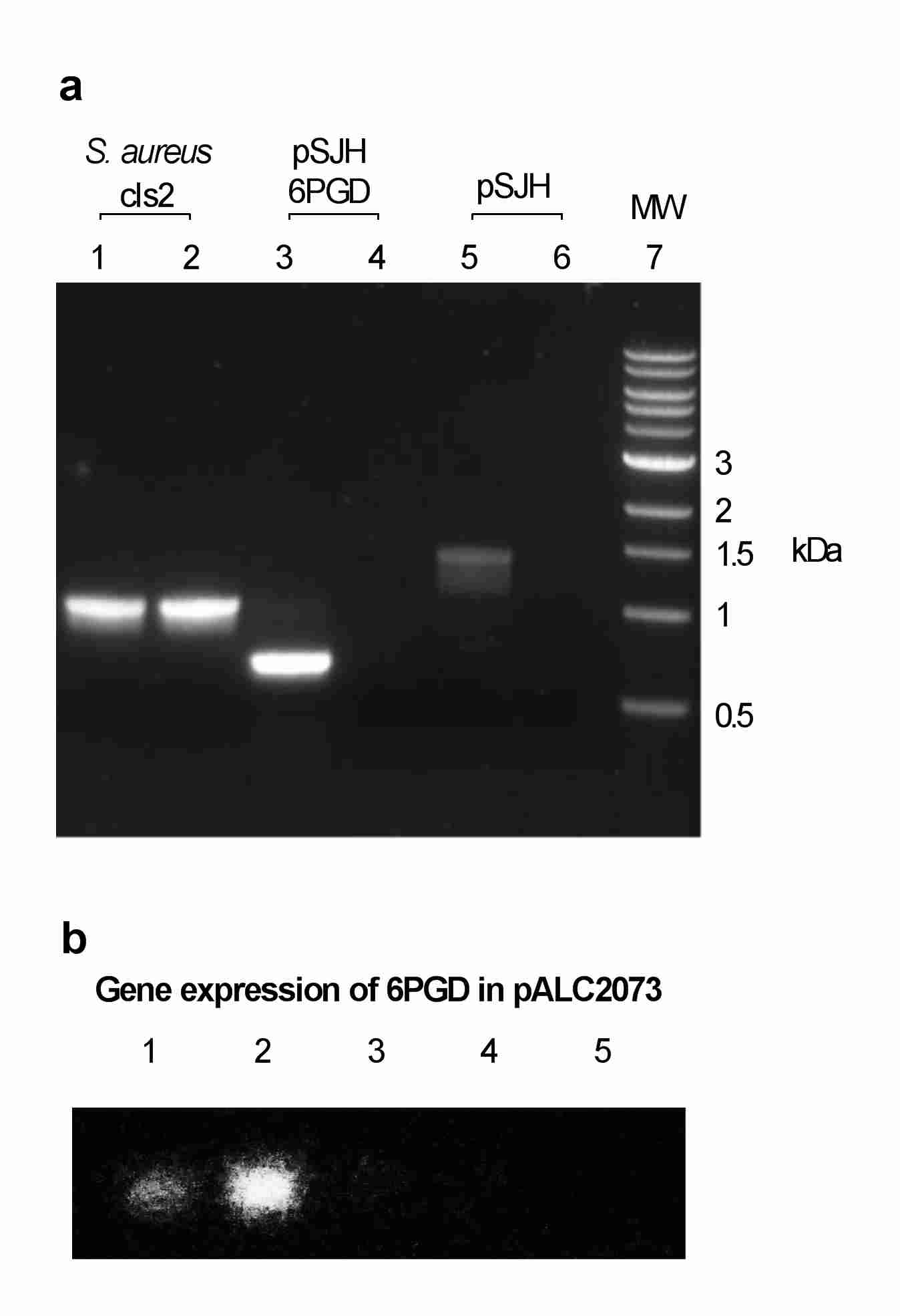
**

**Supplementary Figure 2. Manipulation of *S. aureus* and plasmids. a** Curing removes pSJH101 from *S. aureus* JH1. PCR products of *S. aureus* JH1 on lanes 1, 3 and 5; PCR products of cured *S. aureus* JH1 on lanes 2, 4 and 6. Gel electrophoresis of PCR products show the detection *cls2* (1 kb) in lanes 1 and 2 (positive control to confirm presence of *S. aureus* chromosomal DNA, detection of pSJH101 (700 bp) only in lane 3, and detection of pSJH101 derived 6PGD (1.5 kb) only in lane 5. **b** Gene expression of 6PGD in pALC2073. Gel electrophoresis of PCR products on cDNA from *S. aureus* RN4220 containing pALC2073 with 6PGD cultured without tetracycline (lane 1), *S. aureus* RN4220 containing pALC2073 with 6PGD cultured with tetracycline (lane 2), *S. aureus* RN4220 containing pALC2073 alone cultured with tetracycline (lane 3), *S. aureus* RN4220 chromosomal DNA (lane 4), and water (negative control) (lane 5), showing expression of 6PGD in cultured *S. aureus* RN4220 containing pALC2073 with 6PGD, which is enhanced when cultured with tetracycline. The full gel for panel **a** is provided as a Source Data file**.**

**Supplementary Figure 3**

**Supplementary Figure 3. Delineation of the core and critical amino acids of MPO_409-428_ in BALB/c and HLA-DR15 transgenic (Tg) mice.** Corresponding residues defined in C57BL/6 (I-A^b^) mice^1^, with the critical amino acids in bold, are also shown. **a** BALB/c (*n* = 4) and HLA-DR15 Tg (*n* = 4) mice were immunized with MPO_409-428_, then draining lymph node cells restimulated with shortened 16-mers and 12-mers in IFN-γ and IL-17A ELISPOTs. **b** BALB/c (*n* = 4) and HLA-DR15 Tg mice (*n* = 4) were immunized with MPO_413-428_, then draining lymph node cells restimulated with overlapping 12-mers using a [^3^H]-thymidine proliferation assay. Letters in red represent the core amino acids. **c** BALB/c (*n* = 5) and HLA-DR15 Tg (*n* = 5) mice were immunized with MPO_415-425_ (+: KLYQEARKIVG), then draining lymph node cells restimulated with alanine substituted peptides in sequential order using a [^3^H]-thymidine proliferation assay. For example, K = ALYQEARKIVG and L = KAYQEARKIVG. Alanine itself was substituted for serine. The negative control (-) was OVA_323-339_. Error bars represent the mean ± s.e.m. **P*<0.05, ***P*< 0.01, ****P*<0.001 by Kruskal-Wallis test relative to the response to the immunizing antigen. Source data are provided as a Source Data file**.**

**Supplementary Figure 4**

**
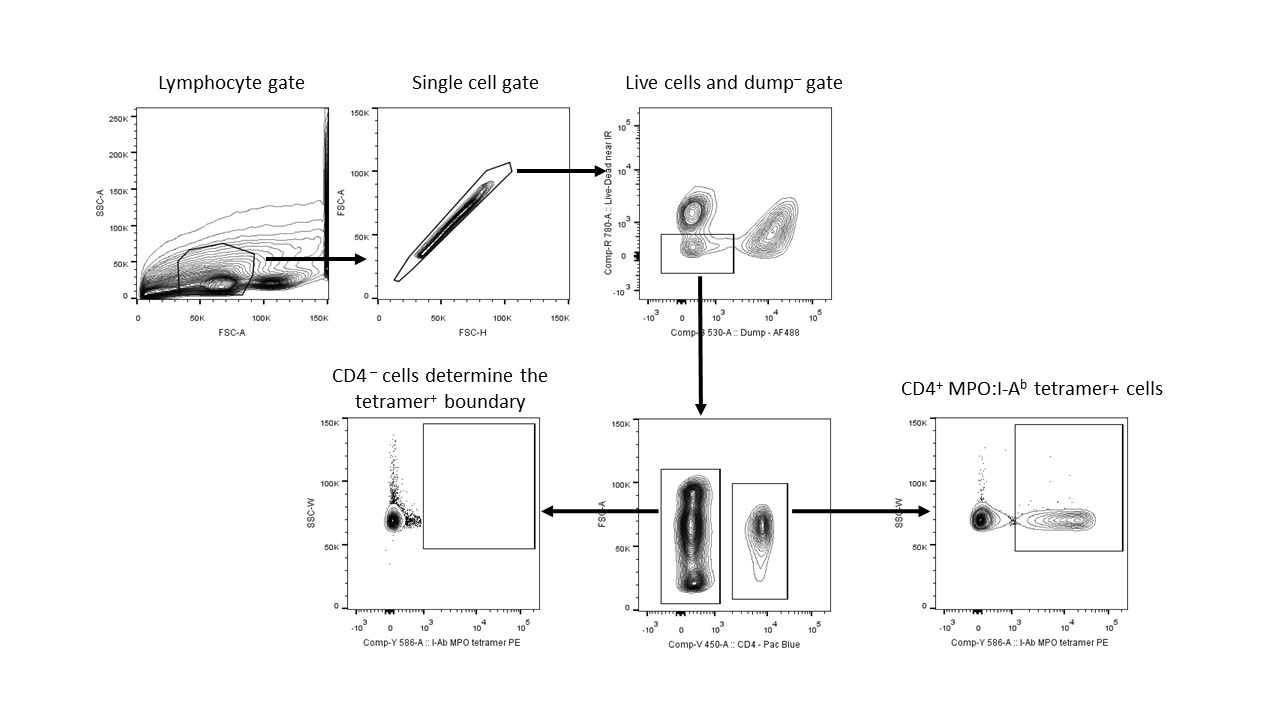
**

**Supplementary Figure 4. Gating strategy for the identification of MPO:I-A^b^ tetramer positive cells.** This Figure relates to the data shown in Figure 2b.

**Supplementary Table 1. Peptides derived from microbes reported to colonize humans with closest sequence homology to the immunodominant MPO T cell epitope**

| Organism | Sequence | Protein | BLAST MAX score | Ref |
| --- | --- | --- | --- | --- |
| *Homo sapiens* | _441_RLYQEARKIVG_451_ | Myeloperoxidase | 38.4 | ^1^ |
| *Treponema vincentii* | _173_RLYKEARKIQK_183_ | Hypothetical protein | 28.2 | ^2^ |
| *Aspergillus fumigatus* | _831_RWYQEARKIIF_841_ | HEAT repeat protein | 27.4 | ^3^ |
| *Helicobacter pylori* | _169_ELYEEARKIIN_179_ | RNA polymerase factor sigma-54 | 27.4 | ^4^ |
| *Bacteroides sp. D22* | _110_TFYQEARKIID_120_ | Chloramphenicol O-acetyltransferase | 27.4 | * |

Ref, reference; * Human Microbiome U54 initiative, Broad Institute (broadinstitute.org)

**Supplementary Table 2. Characteristics of the Monash Cohort of patients with active first presentation MPO-AAV**

| Number^a^ | Age^b^ | Gender | Phenotype^c^ | MPO-ANCA Titer^d^ |
| --- | --- | --- | --- | --- |
| 1 | 70 | F | MPA | 193 |
| 2 | 62 | M | MPA | >200 |
| 3 | 72 | M | MPA | >200 |
| 4 | 65 | M | MPA | 117 |
| 5 | 72 | M | MPA | 70 |
| 6 | 72 | F | MPA | 96 |
| 7 | 50 | M | GPA | 62 |
| 8 | 64 | M | RLV | 173 |
| 9 | 61 | F | MPA | 117 |
| 10 | 66 | M | MPA | 145 |
| 11 | 55 | F | MPA | >200 |
| 12 | 49 | M | MPA | 186 |
| 13 | 52 | M | MPA | >200 |
| 14 | 73 | M | GPA | 194 |
| 15 | 50 | M | MPA | >200 |

^a^ In patients 1-4 the IgG was purified from first plasmapheresis session effluent, in patients 5-15 IgG was from sera taken within two weeks of diagnosis

^b^ Age at diagnosis

^c^ GPA, granulomatosis with polyangiitis; MPA, microscopic polyangiitis; RLV, renal limited vasculitis

^d^ Measured at Monash Health Pathology, Diagnostic Immunology by ELISA (units AU/ml), upper limit of normal 20, all patients were pANCA positive by indirect immunofluorescence.

**Supplementary Table 3. Variant peptide sequences of 6-phosphogluconate dehydrogenase in other strains of *S. aureus***

| *Staphylococcus aureus* variants | Sequence | BLAST MAX score |
| --- | --- | --- |
| JH1 (pSJH101) | _397_TDYQEALRDVVA_408_ | 41.4 |
| Variant 1 | _397_TDYQDALRDVVA_408_ | 38.8 |
| Variant 2 | _397_TNYQEALRDVVA_408_ | 38.8 |
| Variant 3 | _397_TEYQDALRDVVA_408_ | 36.3 |
| Variant 4 | _397_TNYQDALRDVVA_408_ | 36.3 |
| Mammalian 6PGD | _379_DNCQDSWRRVIS_390_ | No similarity |

**Supplementary References**

1 Ooi, J. D. *et al.* The immunodominant myeloperoxidase T-cell epitope induces local cell-mediated injury in antimyeloperoxidase glomerulonephritis. *Proc. Natl. Acad. Sci. USA* **109**, E2615-2624 (2012).

2 Mangan, D. F., Laughon, B. E., Bower, B. & Lopatin, D. E. In vitro lymphocyte blastogenic responses and titers of humoral antibodies from periodontitis patients to oral spirochete isolates. *Infect. Immun.* **37**, 445-451 (1982).

3 Fedorova, N. D. *et al.* Genomic islands in the pathogenic filamentous fungus Aspergillus fumigatus. *PLoS Genet.* **4**, e1000046 (2008).

4 Marshall, B. J. & Warren, J. R. Unidentified curved bacilli in the stomach of patients with gastritis and peptic ulceration. *Lancet* **1**, 1311-1315 (1984).
