# Supplementary material for: A plasmid-encoded peptide from Staphylococcus aureus induces anti-myeloperoxidase nephritogenic autoimmunity
Source: Nat Commun. 2019 Jul 29;10:3392. doi: 10.1038/s41467-019-11255-0 (PMC6662820; doi:10.1038/s41467-019-11255-0)
Supplement: Supplementary file 3 — Reporting Summary [file 41467_2019_11255_MOESM3_ESM.pdf]

nature research

Corresponding author(s): NCOMMS-18-16076. Corresponding Author: A. Richard Kitching

Reporting Summary

Nature Research wishes to improve the reproducibility of the work that we publish. This form provides structure for consistency and transparency in reporting. For further information on Nature Research policies, see Authors & Referees and the Editorial Policy Checklist.

Statistical parameters

When statistical analyses are reported, confirm that the following items are present in the relevant location (e.g. figure legend, table legend, main text, or Methods section).

n/a	Confirmed	
☐	☒	The exact sample size (n) for each experimental group/condition, given as a discrete number and unit of measurement
☐	☒	An indication of whether measurements were taken from distinct samples or whether the same sample was measured repeatedly
☐	☒	The statistical test(s) used AND whether they are one- or two-sided<br>*Only common tests should be described solely by name; describe more complex techniques in the Methods section.*
☒	☐	A description of all covariates tested
☒	☐	A description of any assumptions or corrections, such as tests of normality and adjustment for multiple comparisons
☒	☐	A full description of the statistics including central tendency (e.g. means) or other basic estimates (e.g. regression coefficient) AND variation (e.g. standard deviation) or associated estimates of uncertainty (e.g. confidence intervals)
☐	☒	For null hypothesis testing, the test statistic (e.g. F, t, r) with confidence intervals, effect sizes, degrees of freedom and P value noted<br>*Give P values as exact values whenever suitable.*
☒	☐	For Bayesian analysis, information on the choice of priors and Markov chain Monte Carlo settings
☒	☐	For hierarchical and complex designs, identification of the appropriate level for tests and full reporting of outcomes
☒	☐	Estimates of effect sizes (e.g. Cohen's d, Pearson's r), indicating how they were calculated
☐	☒	Clearly defined error bars<br>*State explicitly what error bars represent (e.g. SD, SE, CI)*

Our web collection on statistics for biologists may be useful.

Software and code

Policy information about availability of computer code

Data collection	No software was used
Data analysis	GraphPad Prism

For manuscripts utilizing custom algorithms or software that are central to the research but not yet described in published literature, software must be made available to editors/reviewers upon request. We strongly encourage code deposition in a community repository (e.g. GitHub). See the Nature Research guidelines for submitting code & software for further information.

Data

Policy information about availability of data

All manuscripts must include a data availability statement. This statement should provide the following information, where applicable:
- Accession codes, unique identifiers, or web links for publicly available datasets
- A list of figures that have associated raw data
- A description of any restrictions on data availability

The data that support the findings of this study are available from the corresponding author upon reasonable request.

Field-specific reporting

Please select the best fit for your research. If you are not sure, read the appropriate sections before making your selection.

☒ Life sciences ☐ Behavioural & social sciences ☐ Ecological, evolutionary & environmental sciences

For a reference copy of the document with all sections, see nature.com/authors/policies/ReportingSummary-flat.pdf

Life sciences study design

All studies must disclose on these points even when the disclosure is negative.

Sample size	In previous work in defining immuodominant MPO T cell epitopes a sample size of n=4-6 per group has been sufficient to determine bioloigcla relevance. This approach was also used in the current studies.
Data exclusions	No data were excluded from experiments.
Replication	The majority of studies within the work were replicated, as indicated in the manuscript. Some studies were interdependent. As an example. the results of the studies using whole S. aureus are concordant and reinforce each other - they tested the same hypothesis using the same model but in a different manner.
Randomization	Mice were randomly allocated to experimental groups.
Blinding	Histological assessment was performed in a blinded fashion on coded slides.

Reporting for specific materials, systems and methods

Materials & experimental systems

n/a	Involved in the study
☐	☒ Unique biological materials
☐	☒ Antibodies
☐	☒ Eukaryotic cell lines
☒	☐ Palaeontology
☐	☒ Animals and other organisms
☐	☒ Human research participants

Methods

n/a	Involved in the study
☒	☐ ChIP-seq
☐	☒ Flow cytometry
☒	☐ MRI-based neuroimaging

Unique biological materials

Policy information about availability of materials

Obtaining unique materials	Unique materials, for example constructs for 6PGD plasmid sequences and MPO/I-Ab tetramers will be made available if possible, on request.

Antibodies

Antibodies used	CD4-Pacific Blue, anti-mouse CD11c, CD11b, F4/80, CD8a, B220-Alexa Fluor 488:<br>Anti-mouse IFN-g and anti-mouse IL-17A<br>anti-mouse IgG HRP; indirect immunofluorescence chicken anti-mouse AF 488.<br>clones GK1.5, 53-6.7, FA/11, RB6-8C5<br>Anti-mouse IFN-g, TNF, IL-17A and IL-6
Validation	Flow cytometry: anti-mouse CD4-Pacific Blue (Biolegend, #100531), and anti-mouse CD11c, CD11b, F4/80, CD8a, B220-Alexa Fluor 488 (Biolegend, #117311, #101217, #123120, #100723, #103225). These are standard monoclonal antidies specific for their antigenic targets and validated on multiple occasions.<br>For ELISPOT: anti-mouse IFN-g and anti-mouse IL-17A (eBioscience, #551216, #554410, #555068 and #555067) are established, validated and specific antibodies.<br>Secondaries to detect MPO-ANCA: anti-mouse IgG HRP (Amersham); indirect immunofluorescence chicken anti-mouse AF 488.<br>For tissue immunostaining: clones GK1.5 (ATCC), 53-6.7 (BioXcell) FA/11, RB6-8C5 (grown in house). These are standard, validated, specific monoclonal antibodies specific for their antigenic targets used for immunostaining on multiple occasions.

	Anti-mouse IFN-g, TNF, IL-17A and IL-6 (Cytometric Bead Array, BD Biosciences, detailed in Instruction Manual #560485)

Eukaryotic cell lines

Policy information about cell lines

Cell line source(s)	High Five insect cells (Trichoplusia ni BTI-Tn-5B1-4 cells), and Sf9 and Sf21 insect cells (Spodoptera frugiperda) were purchased from Invitrogen to generate MPO:I-Ab tetramers and recombinant murine MPO.
Authentication	By Invitrogen
Mycoplasma contamination	Cell lines were not tested for Mycoplasma
Commonly misidentified lines (See ICLAC register)	None

Animals and other organisms

Policy information about studies involving animals; ARRIVE guidelines recommended for reporting animal research

Laboratory animals	C57BL/6 mice and BALB/c mice were from the Monash Animal Research Platform, Monash University). Mpo-/- mice and HLA-DR15 transgenic mice were bred at the Monash Medical Centre Animal Facility (MMCAF), Monash Medical Centre, Clayton. Mice were housed in the SPF facilities at MMCAF. Experiments were conducted in male mice aged 6-10 weeks of age.
Wild animals	The study did not involve wild animals.
Field-collected samples	The study did not involve samples collected from the field.

Human research participants

Policy information about studies involving human research participants

Population characteristics	All patients had a confirmed diagnosis of ANCA-associated vasculitis (AAV), fulfilling the Chapel Hill Consensus Conference definitions of AAV. All patients were confirmed positive for either PR3-ANCA or MPO-ANCA by capture ELISA and indirect immunofluorescence on ethanol fixed neutrophils
Recruitment	Participants receiving clinical care at University Medical Center Groningen, The Netherlands and Monash Health, Melbourne, Australia were approached by clinicians involved in their clinical care.

Flow Cytometry

Plots

Confirm that:

☒ The axis labels state the marker and fluorochrome used (e.g. CD4-FITC).

☒ The axis scales are clearly visible. Include numbers along axes only for bottom left plot of group (a 'group' is an analysis of identical markers).

☒ All plots are contour plots with outliers or pseudocolor plots.

☒ A numerical value for number of cells or percentage (with statistics) is provided.

Methodology

Sample preparation	To determine the in vivo expansion of MPO specific cells, mice were first immunized with 10 ug of peptide emulsified in FCA subcutaneously base of tail, then, 7 days later, the inguinal, axillary, brachial, cervical, mesenteric, and periaortic lymph nodes and spleen were harvested . Single cell suspensions were made by mechanically pushing lymph nodes and spleen through a 70um mesh.
Instrument	BD LSRFortessa X-20, at the Monash Health Translation Precinct
Software	FlowJo version 10
Cell population abundance	The abundance of the relevant cell population (tetramer+ cells) in the post-magnetic bead isolation ranged from 1 cell per million (OVA immunised) to 165 cells per million (MPO immunised).

Gating strategy

Gate 1: Lymphocyte gate based on FSC-A and SSC-A
Gate 2: Single cell gate based on FSC-A and FSC-H
Gate 3: Live cells not expressing CD11c, CD11b, F4/80, CD8a, and B220 (boundary determined based on FMO controls)
Gate 4: CD4+ cells (boundary based on FMO control)
Gate 5: Tetramer positive cells (boundary based on CD4- population)

☒ Tick this box to confirm that a figure exemplifying the gating strategy is provided in the Supplementary Information.
